# Editorial: Recent Advances in Biocatalysis: Focusing on Applications of These Processes

**DOI:** 10.3389/fbioe.2022.844741

**Published:** 2022-02-17

**Authors:** Immacolata Serra, Lorena Wilson, Andrés R. Alcántara

**Affiliations:** ^1^ Department of Biotechnology and Biosciences, University of Milano-Bicocca, Milan, Italy; ^2^ Pontificia Universidad Católica de Valparaíso, Valparaíso, Chile; ^3^ Department of Chemistry in Pharmaceutical Sciences (QUICIFARM), Pharmacy Faculty, Complutense University of Madrid (UCM), Madrid, Spain

**Keywords:** biocatalysis, biotransformations, enzymes, whole-cell biocatalysis, enzyme engineering, process optimization

Applied biocatalysis, that is, the use of biocatalysts (as whole cells or isolated enzymes, either in their natural state or chemically and/or genetically modified) is undoubtedly a very powerful tool for the practical implementation of sustainable industrial processes with maximum resource utilization and minimum waste generation, inside the context of a circular bio-based economy. In such a scenario, the Research Topic *Recent Advances in Biocatalysis: Focusing on Applications of these Processes* is presenting very exciting examples illustrating the broad applicability of biocatalysis.

Thus, Wang et al. reported how the combined use of a biocatalyzed step (whole cells from fungi) and an extraction of phenolic compounds is leading to an effective production of Iturin A (a cyclo-lipopeptide generally applied in the biological control of plant diseases) starting from rapeseed meal (RSM), a major by-product of oil extraction from rapeseed. This is a nice example illustrating the combination of biological pretreatment with chemical extraction and biotransformations from bio-based residues to fine chemicals, according to the concept of biorefinery.

Capusoni et al. screened a collection of 28 yeasts isolated from different environments, marine and terrestrial, and identified new phytase activities in yeasts such as *C. jadinii*, *K. marxianus*, and *T. delbrueckii*. In particular, *C. jadinii* CJ2 was the best producer of both secreted and cell-bound phytase which showed a remarkable activity at high temperature and acidic pH. These characteristics make the *C. jadinii* enzyme a promising candidate for feed/food-related processes for phytic acid degradation.

On the other hand, Menegatti and Žnidaršič-Plazl presented the development of a microbioreactor between two plates using an amine transaminase immobilized (together with the cofactor pyridoxal phosphate (PLP)) in a porous copolymeric hydrogel matrix. This experimental array did not require either organic solvents or any additional polycationic polymers for successful PLP retention, and allowed an enhanced enzymatic stability over a wider pH and temperature range compared to the free enzyme, as it was possible to retain 92% of the initial productivity after 10 days of continuous operation, even at 50°C.

One of the current drawbacks of commercially available enzymes is derived from their mesophilic origin, which limits the optimal ranges of temperature and pH (i.e., between 20 and 45°C, neutral pH) that can be used. Thus, for industrial applications, an efficient solution is to use enzymes from extremophiles, which display higher activity, stability, and robustness compared to mesophilic counterparts, therefore allowing the development of biocatalysis at nonstandard conditions. In this sense, Espina et al. have reported a stepwise strategy for the development (cloning, over-expression, and downstream processing) and industrial production of three different extremozymes (a psychrotolerant catalase, a thermoalkaliphilic laccase, and a thermophilic amine-transaminase) isolated from several extreme environmental conditions in Antarctica, thus expanding the arsenal of available biocatalysts.

A different strategy to obtain biocatalysts with improved properties is represented by protein engineering. In this frame, Liu et al. described the identification of the key residues controlling the enantioselectivity of esterase Est924 belonging to the bSHL family and previously identified from a metagenomic library of bamboo root soil. In addition, rational engineering was applied to this enzyme, resulting in variants switching enantioselectivity (from (*R*) to (*S*) toward different ethyl 2-arylpropionates), including some non-steroidal anti-inflammatory drugs such as ketoprofen, naproxen, and ibuprofen. Finally, using whole-cell biocatalysts harboring one of the variants (M3, I202F/A203W/G208F), the kinetic resolution of ketoprofen and naproxen ethyl esters to the corresponding (*S*)-carboxylic acids was achieved with 95 and 96% ee, respectively, and with good biocatalyst recyclability.

A similar approach was used by Pan et al. to obtain a variant of a keratinase from *Pseudomonas aeruginosa* (KerPA) with increased activity and thermostability for industrial application. The best selected variant, Y21pBpF/Y70pBpF/Y114pBpF, obtained through the insertion of non-canonical amino acids, showed, compared to the wild-type enzyme, an increased activity and half-life. This paper is an important example of how the incorporation of non-canonical amino acids into a protein may greatly expand the range of new possible interactions between the side chains of the residues and, consequently, promoting the creation of new molecular structures that result in enhanced physical, chemical, and biological properties of the mutated enzymes.

